# Supplementary material for: Progress toward SHAPE Constrained Computational Prediction of Tertiary Interactions in RNA Structure
Source: Noncoding RNA. 2021 Nov 5;7(4):71. doi: 10.3390/ncrna7040071 (PMC8628965; doi:10.3390/ncrna7040071)
Supplement: Supplementary file 1 [file ncrna-07-00071-s001.zip › ncrna-1422092-sup.fig.pdf]

SUPPLEMENTARY FIGURES:

Supplementary figure S1

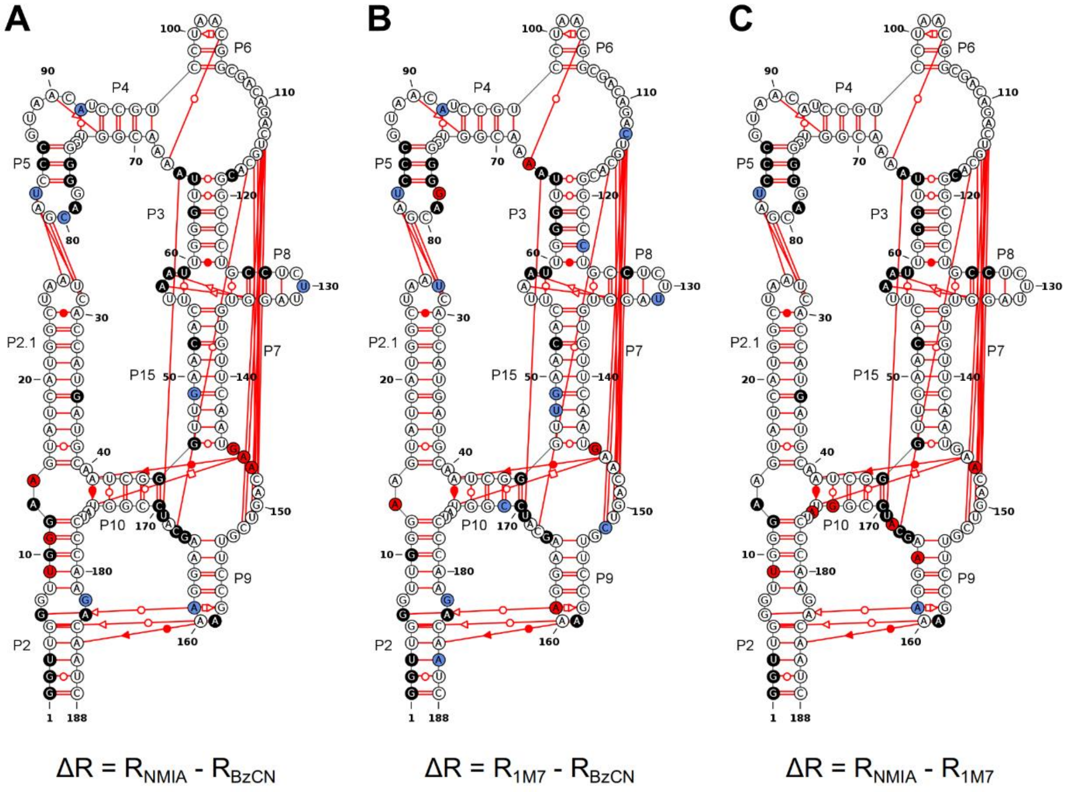

S1: Comparison of the reactivity obtained with the three probes

DiLCrz structure was probed with 1M7, BzCN and NMIA at 37°C in 40mM HEPES pH 7.5, 500mM KCl and 5 mM $MgCl_2$ and reactivity profiles were compared side-by-side. Nucleotides are coloured according to the indicated difference in reactivity $\Delta R = R_{probe\_1} - R_{probe\_2}$. Nucleotides in blue indicate a significant negative ΔR while nucleotides in red indicate a significant positive ΔR.

Supplementary figure S2

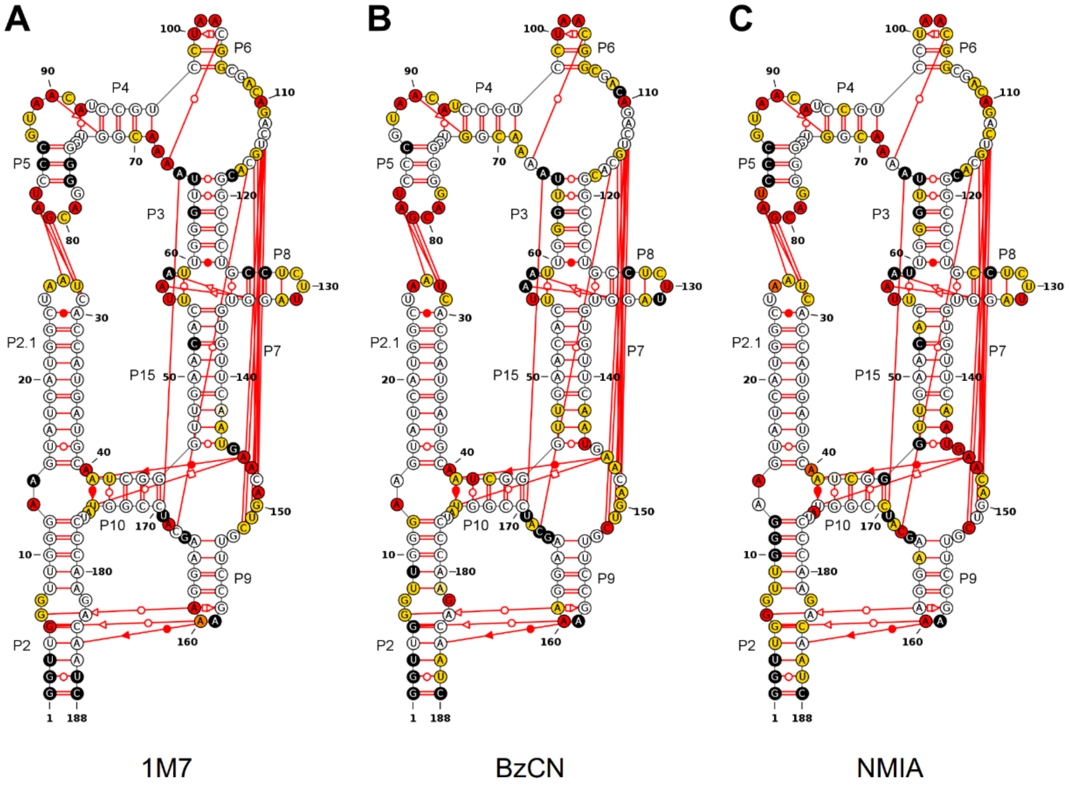

S2: Reactivity profiles in absence of Mg^{2+}

DiLCrz structure was probed with 1M7, BzCN and NMIA at 37°C in 40mM HEPES pH 7.5 and 500mM KCl. Nucleotides are coloured according to their reactivity. White: low reactivity (0 to 0.4), yellow : moderate reactivity (0.4 to 0.7), red: high reactivity (over 0.7), black: undetermined reactivity.

Supplementary figure S3

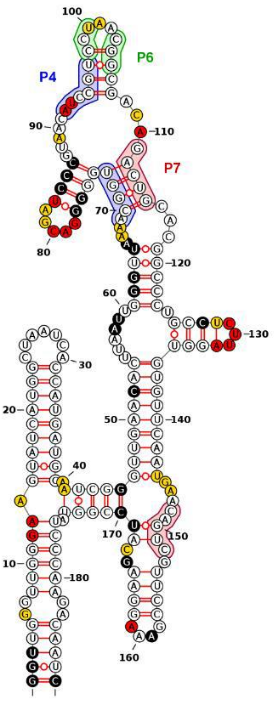

S3: Model obtained with RNAfold and RNAstructure using 1M7 reactivities as soft constraints

DiLCrz structure was probed with 1M7 at 37°C in 40mM HEPES pH 7.5, 500mM KCl and 5 mM $MgCl_2$. Either RNAfold or RNAstructure were run with 1M7 reactivities as soft constraints. Shown is the minimum free energy structure model output by both software with the nucleotides coloured according to their 1M7 reactivity. White: low reactivity (0 to 0.4), yellow : moderate reactivity (0.4 to 0.7), red: high reactivity (over 0.7), black: undetermined reactivity. Pairings that RNAfold and RNAStructure failed to predict are boxed in blue (P4), green (P6) and red (P7 pseudoknot).

Supplementary figure S4

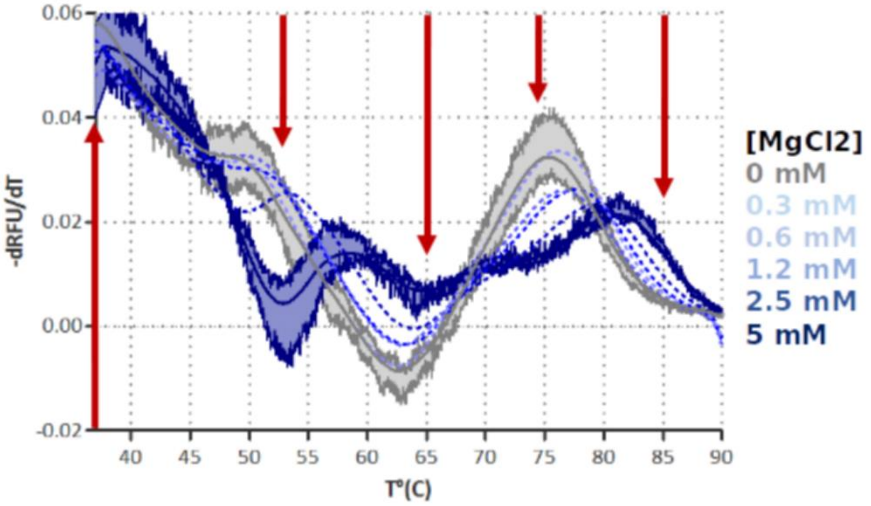

S4: Melting profiles of the wild-type DiLCrz with error bars

Melting curves of DiLCrz obtained with RiboGreen in the presence of increasing amounts of magnesium. Gray: absence of magnesium; light blue to dark blue: 0, 0.3, 0.6, 1.2, 2.5 and 5 mM MgCl2, respectively. Fluorescence derivatives were averaged from three independent experiments. For 0 and 5 mM $MgCl_2$, the highlighted areas indicate the standard error of the mean. The red arrows indicate the temperatures at which probing was conducted.

Supplementary figure S5

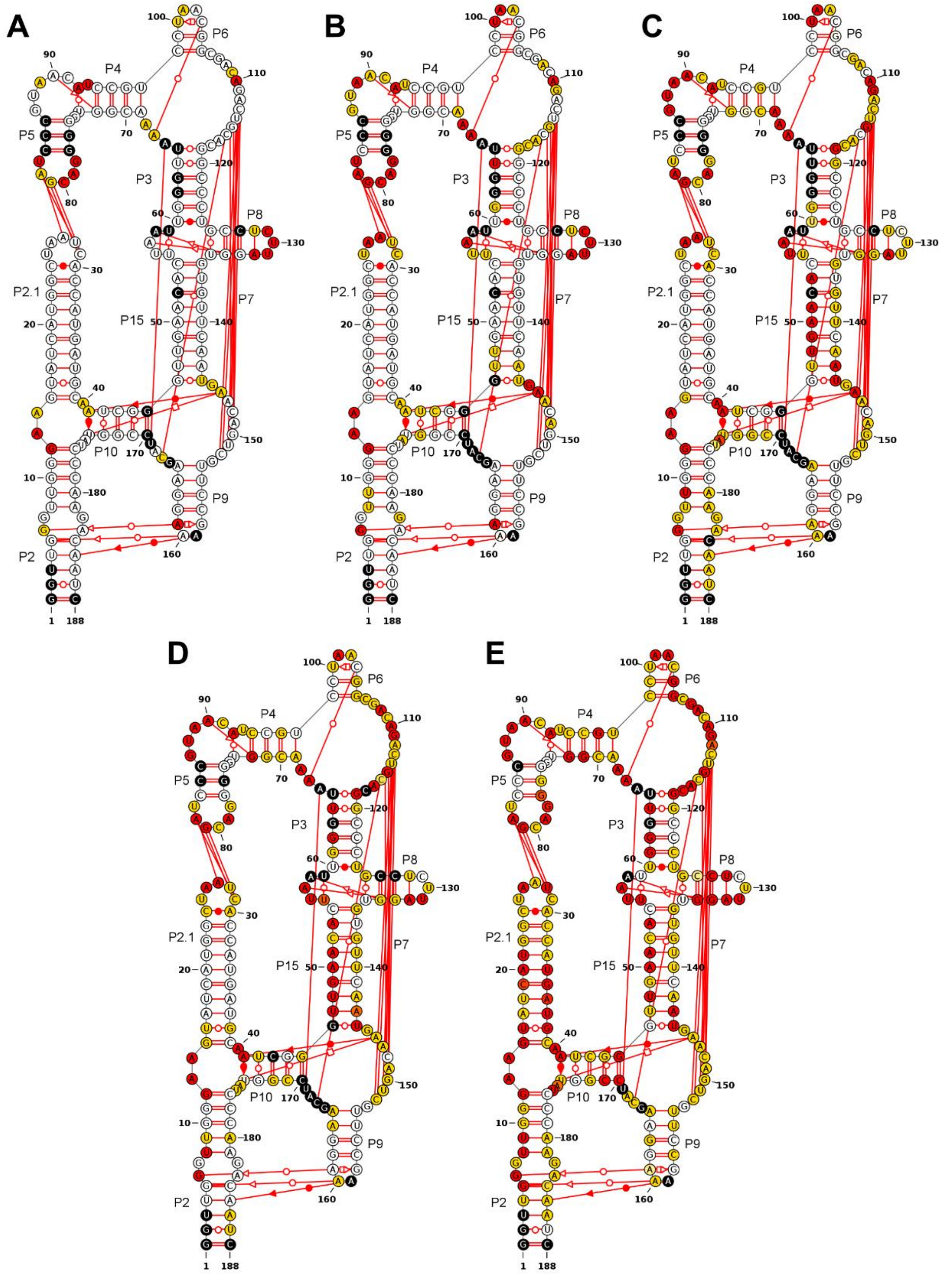

S5: 1M7 probing profiles at different temperature

DiLCrz was probed with 1M7 in 40mM HEPES pH 7.5, 500mM KCl and 5 mM $MgCl_2$ at 37°C (A), 53°C (B),

65°C (C), 74°C (D) and 85°C (E). Reactivity profiles were pasted over DiLCrz secondary structure. White: low reactivity (0 to 0.4), yellow: moderate reactivity (0.4 to 0.7), red: high reactivity (over 0.7), black: undetermined reactivity.

Supplementary figure S6

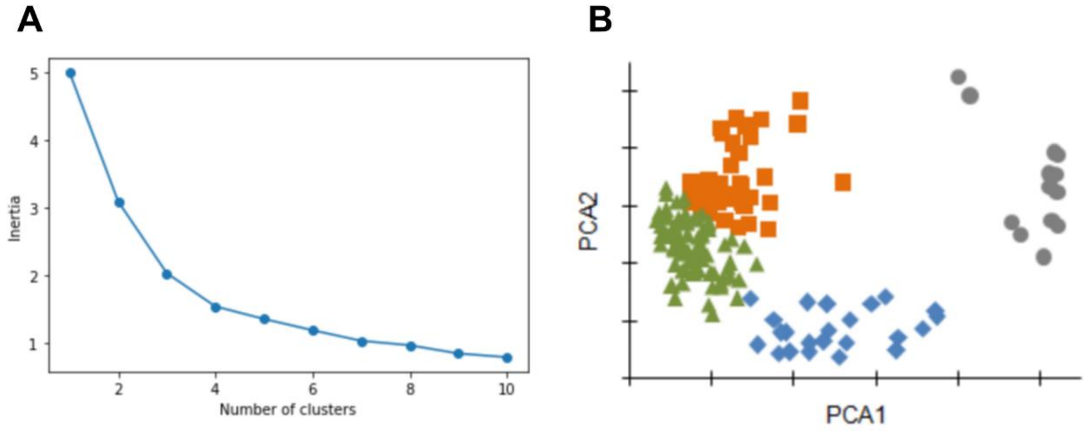

S6: Clustering nucleotides according to 1M7 probing profiles at different temperature

A. Principal component analysis of the reactivities obtained at different temperatures suggests that nucleotides can be clustered. Dots are coloured according to their assigned cluster as in figure 2.

B. The elbow method was used to determine the optimal number of clusters. KMeans algorithm was run with each indicated number of clusters to calculate the distortion, or within-cluster sum of squared errors. The change in distortion becomes less pronounced above 4 clusters, suggesting that 4 is the optimal cluster number.

Supplementary figure S7

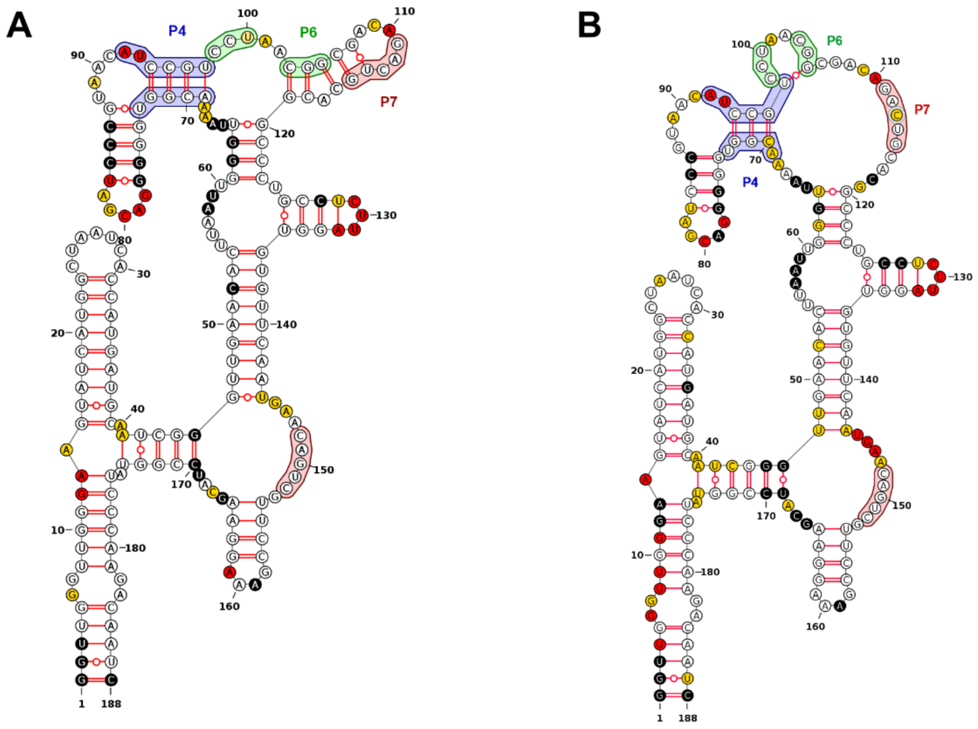

S7: Secondary structure models obtained with IPANEMAP. Either 1M7 (**A**) or NMIA (**B**) reactivities obtained in presence of Mg^{2+} were used as soft constraints. Nucleotides are colour-coded according to their reactivity. White: low reactivity (0 to 0.4), yellow : moderate reactivity (0.4 to 0.7), red: high reactivity (over 0.7), black: undetermined reactivity. P4, P5 and P7 are highlighted in colours.
